# Serum chitinase activity prognosticates metastasis of colorectal cancer

**DOI:** 10.1186/s12885-019-5834-7

**Published:** 2019-06-25

**Authors:** Zhangfa Song, Engeng Chen, Jun Qian, Jianbin Xu, Gaoyang Cao, Wei Zhou, Fei Wang, Min Chen, Dengyong Xu, Xiaowei Wang, Xiaotong Hu, Zhenyu Ju, Xuefeng Huang, Xiujun Cai

**Affiliations:** 1 Department of Colorectal Surgery, Sir Run Run Shaw Hospital of Zhejiang University, Hangzhou, 310016 China; 2 Zhejiang Province Key Laboratory of Biological Treatment, Hangzhou, China; 3 Department of colorectal surgery, Xinchang Hospital, Xinchang, China; 4 Key Laboratory of Regenerative Medicine of Ministry of Education, Institute of Aging and Regenerative Medicine, Jinan University, Guangzhou, China; 5 Department of General Surgery, Sir Run Run Shaw Hospital, Zhejiang University, Hangzhou, 310016 China

**Keywords:** Chitinase activity, Colorectal Cancer, Metastasis, Biomarker

## Abstract

**Background:**

This study aimed to evaluate the value of chitinase activity in prognosticating the occurrence of metastasis in and prognosis of patients with colorectal cancer (CRC).

**Methods:**

The chitinase activity in four different groups, namely 335 CRC patients without distant metastasis at their first visit (Group 1), 51 patients with CRC having synchronous liver metastasis (Group 2), 100 healthy age-matched controls (Group 3) and 40 patients with liver cancer (Group 4), were assayed using an enzyme-linked immunosorbent assay. The Cox proportional hazards ratio model and Kaplan–Meier curve were used to identify the association between chitinase activity and the clinical outcome of CRC patients without metastasis in the training set and testing set at their first visit. An in vitro Transwell experiment was performed to evaluate the migration of colon cancer cells.

**Results:**

Patients with high chitinase activity had a significantly higher metastasis risk than those with low chitinase activity in the training and testing sets during follow-up, both at stage I/II and stage III. Further, multivariate analysis revealed that chitinase activity was an independent risk factor prognosticating liver metastases (*P* = 0.001). The combination of chitinase activity and lymph node metastasis status increased the accuracy of the prognosis of liver metastases after radical resection (*P* = 0.454E-011). In addition, chitinase promoted CRC cell migration in vitro.

**Conclusions:**

Chitinase activity can prognosticate the occurrence of metastasis in patients with CRC. Moreover, the combination of chitinase activity and N stage increased the power of prognosticating the occurrence of metastasis. Inhibiting chitinase activity may serve as a new strategy to treat metastases of CRC.

**Electronic supplementary material:**

The online version of this article (10.1186/s12885-019-5834-7) contains supplementary material, which is available to authorized users.

## Background

Colorectal cancer (CRC) is one of the most common cancers and the second leading cause of cancer-related mortality worldwide. The majority of CRC cases have regional and distant status, with 5-year survival rates of 71.2 and 13.5%, respectively [1]. It is obvious that distant metastasis is the main cause of death in patients with CRC. The liver is the most commonly involved organ in CRC, with approximately 21% of CRC patients having synchronous liver metastasis [1, 2]. Furthermore, liver metastasis occurs in 30–40% patients with CRC during the disease course [3]. Chemotherapy and biological agents significantly improve disease-free survival and overall survival (OS).

Unfortunately, less than one-third of liver metastases in CRC patients are potentially resectable at the time of diagnosis. A useful and stable biomarker to prognosticate the early diagnosis of liver metastases is urgently needed to overcome this problem.

We previously identified several inflammatory biomarkers (chitinase activity, stathmin level, elongation factor 1 alpha protein level, and *N*-acetyl-glucosaminidase activity) of biological aging [4]. Available evidence shows a relationship between inflammation and cancer initiation and progression. Chitinase-3-like protein 1 (CHI3L1), a member of the chitinase family, has been implicated in several cancers, such as glioblastoma, breast cancer, and ovarian cancer [5–7]. These studies support the role of chitinase-like proteins in tumor cell proliferation, angiogenesis, inflammation, invasion, and metastasis [8]. However, no study has reported a correlation between blood chitinase activity and CRC.

The present study investigated 386 patients with CRC for 2–7 years and found that chitinase activity in blood could prognosticate the occurrence of metastasis in patients with CRC in the training and testing sets, indicating that chitinase activity could be a powerful biomarker for prognosticating the metastasis of CRC.

## Methods

### Study population and follow-up

This was a retrospective study performed on 335 CRC patients without distant metastasis at their first visit (Group 1) that were enrolled at the Sir Run Run Shaw Hospital (SRRSH), Zhejiang University, between 2008 and 2015. These patients were divided into a training set of 99 patients and a testing set of 236 patients based on a random process. Our aim was to identify the significant prognostic value of chitinase activity from the training set and tested it in the internal testing set. Additionally, 51 patients with CRC having synchronous liver metastasis (Group 2) were enrolled between 2012 and 2015. Moreover, 100 healthy age-matched controls (Group 3) that underwent medical examinations without tumors or major illnesses and 40 patients with liver cancer (Group 4) were analyzed together as a control. After written informed consent when admitted, a single blood puncture at the time of initial diagnosis was performed by our professional biobank team according to the Standard operation procedures (SOP) (Biospecimen collection procedures of SRRSH Biobank, Version 1.0). In brief, using Blood Collection Tube (BD Medical 367,983 Vacutainer® Plus Plastic SST™ Blood Collection Tubes with Polymer Gel for Serum Separation) to take blood sample 3 ml and then transporting to laboratory for centrifugation immediately at room temperature. Next, we placed the blood in a centrifuge set at 4 °C for 10 min at 3000 g. After that, We take the supernatant 1.5 ml carefully to microtube (MCT-200-C, 2 ml, Axygen Scientific Inc.). Next, we stored the microtube in a − 80 °C refrigerator until the start of the experiment. All patients who underwent surgery were diagnosed by pathologists in SRRSH. Patients who received (neo) adjuvant radiotherapy or chemotherapy before surgery were excluded. Further, 19 patients with CRC having synchronous liver metastasis received chemotherapy or radiotherapy after losing the opportunity for radical surgery. The CEA levels of patients with CRC were regularly examined before surgery or other treatments. Written informed consent was obtained from all patients before specimen collection. This study was approved by the ethics committee of SRRSH, Zhejiang University.

Patients were followed up through telephone consultations by the study team who was blinded to the experimental results. Overall survival (OS) and Recurrence free time (RFS) were calculated based on the medical records and the follow-up results. OS was defined as the time from a pathology diagnosis (imaging diagnosis date replaced if no pathology diagnosis) to death due to the tumor. RFS was defined as the time from a pathology diagnosis (imaging diagnosis date replaced if no pathology diagnosis) to the appearance of a new metastasis (pathology diagnosis again or imaging diagnosis). OS and RFS were again calculated based on the follow-up results and medical records. Survival and non-metastasis were censored at the time of the last visit if no death or metastasis occurred, respectively. Together, 386 patients with CRC were followed up. The median (interquartile range) follow-up period was 39 (22.59–68.07) months. At the last follow-up on December 1st, 2015, 93 patients (24.1%) had died and 49 (14.6%) of 335 patients without metastasis at their initial diagnosis had distant metastasis occurrence. Therefore we defined patients with CRC with metachronous metastasis as Group 1A and patients with CRC without metastasis until the last follow-up as Group 1B. Then we performed subgroup analysis of stage-by-stage of cox proportional hazards models analysis for identifying the prognosis factors for metastasis between Group 1A and Group 1B.

### Enzyme-linked immunosorbent assay for detecting Chitinase activity

Chitinase activity was measured by an enzyme-linked immunosorbent assay (ELISA) using the Chitinase Assay Kit (Catalog Number CS1030, Sigma-Aldrich, St. Louis, MO, USA). A substrate (4-methylumbelliferyl *N*-acetyl-β-D-glucosaminide) suitable for detecting exochitinase activity (β-*N*-acetylglucosaminidase activity) was chosen for measuring chitinase activity. The experiment was performed according to the manufacturer’s instructions. The standard solution (standard blank, 5 μg/mL, 10 μg/mL, 20 μg/mL, 25 μg/mL, and 50 μg/mL) was added to a 96-well plate to obtain the standard curve. Next, 2 μL of serum were added to each well according to a preset order. The preset order was set that samples from the four groups proportionally in one 96-well plate to achieve homogenization procedures. Then, 100 μL of the assay buffer and 98 μL of substrate working solutions were added to each well using multichannel pipettes (Eppendorf, Hamburg, Germany). The plate was incubated for 30 min at 37 °C after mixing using a horizontal shaker for 2 min. Next, the reactions were stopped by adding 200 μL of stop solution to each well. Subsequently, the fluorescence was measured in a microplate reader (BioTek Synergy™ 2, Winooski, VT, USA) with at an excitation wavelength of 360 nm and an emission wavelength of 450 nm. Each measurement was performed in triplicate.

### Cell line and culture

Human colon cancer cell lines RKO and SW48 were obtained from the American Type Culture Collection (Manassas, VA, USA). The two cell lines were cultured in Dulbecco’s modified Eagle’s Medium (DMEM, high glucose, Gibco, Thermo Fisher Scientific, Waltham, MA, USA) supplemented with 10% fetal bovine serum (FBS) and 1% streptomycin/penicillin at 37 °C in a 5% CO_2_ atmosphere.

### Transwell migration assay

Cell migration was performed using 24-well Transwell chambers with 8-μm-pore-size polycarbonate membranes (Corning Inc., Corning, NY, USA). After cells grew till the logarithmic phase of growth, they were properly cultured with serum-free DMEM (high glucose) during the 24-h starvation period at 37 °C in a 5% CO_2_ atmosphere. Then, the starving cells were harvested, resuspended at a concentration of 3 × 10^5^ cells in 100 μL with serum-free DMEM (high glucose), and seeded into the upper chambers of a Transwell plate. Chitinase (0 μg, 2 μg, or 4 μg, Catalog Number C6242, Sigma–Aldrich) was added to the upper chambers of the Transwell plate. The lower chambers were filled with 600 μL of DMEM supplemented with 10% FBS. After incubating the cells for 24 h at 37 °C in a 5% CO_2_ atmosphere, the noninvasive cells on the top side of the membrane were removed thoroughly but gently using a cotton swab. The invaded cells on the lower membrane surface were fixed in 20% methanol for 15 min and then stained with 0.1% crystal violet for 30 min. The cells were photographed with four randomly visual fields in each well under a light microscope at 100× magnification (Olympus, Tokyo, Japan) and counted using Image-Pro Plus 6.0 software (Media Cybernetics, Inc., Rockville, MD, USA).

### Statistical analysis

Student’s t-test, Chi-squared test, Mann–Whitney test, Kruskal–Wallis test and one-way analysis of variance (ANOVA) were used to analyze differences of variables among subgroups according to data type and distribution. The Kolmogorov-Smirnov test was used to judge the normality of continuous data distribution. The optimal cutoff point of chitinase activity for metastasis risk prediction in the training set was calculated based on the receiver operating characteristic (ROC) curve and applied in the testing set [9]. The comparisons of ROC curves were used to discriminate the power of the biomarkers in predicting metastasis. Kaplan–Meier analyses with the log-rank test were used to analyze metastasis probability and prognosis. The multivariate Cox proportional hazards analysis was used to identify independent predictors of metastasis. All statistical analyses were performed using SPSS Software V19 (IBM, Armonk, NY, USA), MedCalc V12.7 (MedCalc Software, Ostend, Belgium), and GraphPad Prism V6.0 (GraphPad Software, San Diego, CA, USA). *P* values less than 0.05 in a two-tailed test were considered statistically significant.

## Results

### Chitinase activity correlated with OS in patients with CRC

**Fig. 1:**
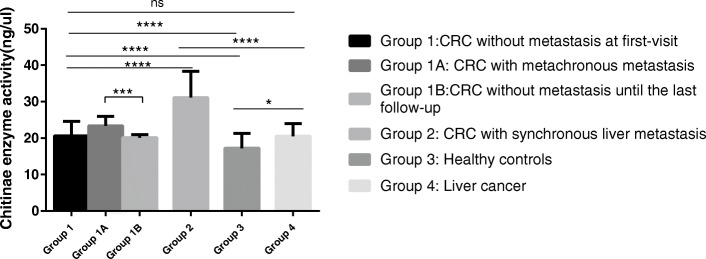
Chitinase activity correlates with metastases in colorectal cancer patients. Bar graph showed that the level of chitinase activity in different groups. Kruskal-Wallis test among Group 1,2,3,4. Mann Whitney test between Group 1A and 1B; Data in bar graph are shown as median with interquartile range; CRC, Colorectal Cancer. *P < 0.05, ***P < 0.001, ****P < 0.0001

**Fig. 2:**
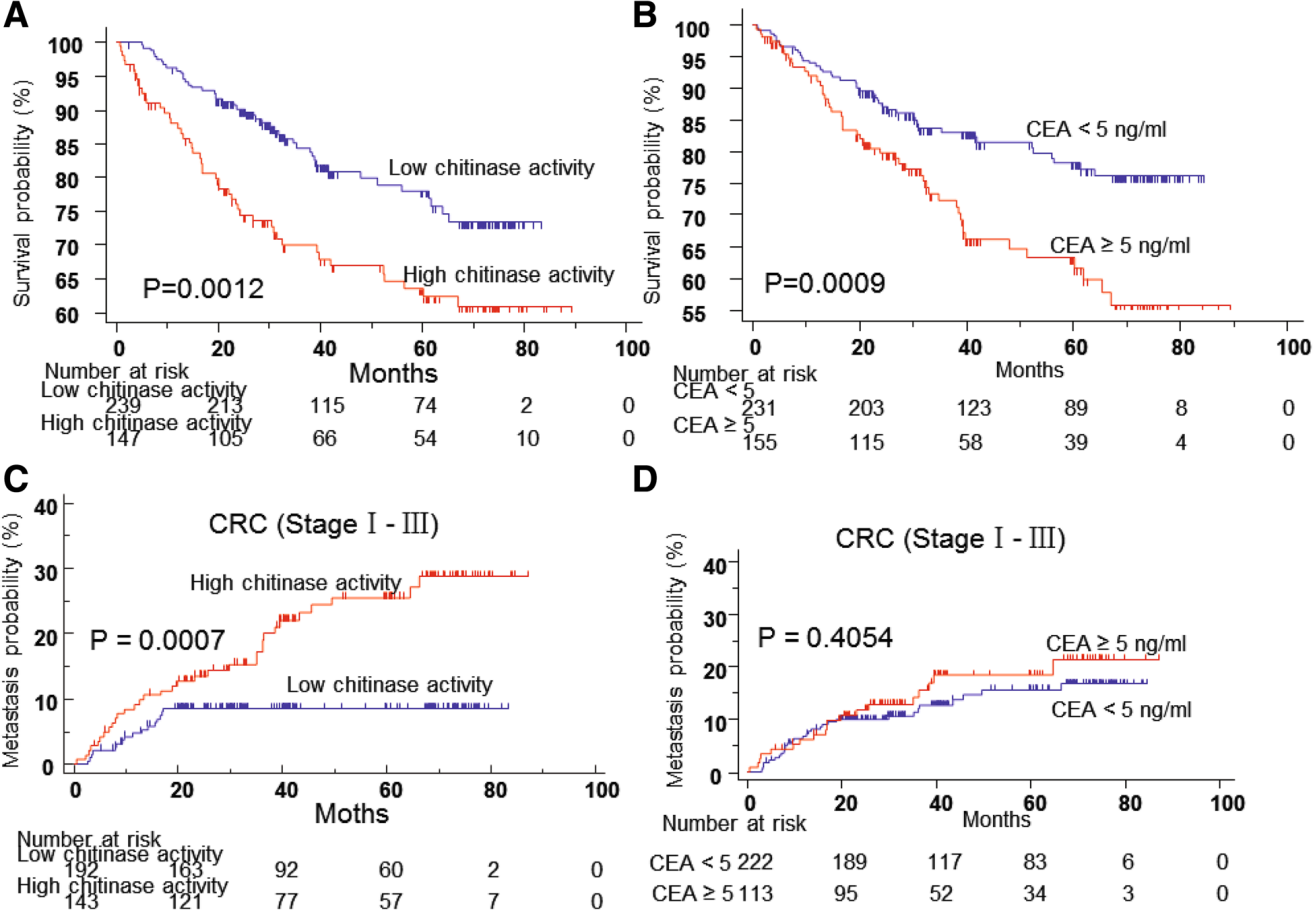
Chitinase activity correlates with metastases and prognosticates metastases of primary colorectal cancer. **a** Kaplan-Meier analysis showed the chitinase activity in serum had significant correlation to survival in colorectal cancer patients. **b** Kaplan-Meier analysis showed CEA level in serum had significant correlation to survival in colorectal cancer patients. **c** Kaplan–Meier curves of metastasis probability of CRC patients without metastasis at first visit in the populations. It showed that patients with high chitinase activity have more metastasis risk compared with those with low chitinase activity. **d** Kaplan–Meier curve showed that CRC patients with high CEA level were no more likely to metastasize compared with CRC patients with low CEA level at Stage І-III (*p* = 0.4054). Log-rank test were performed in all figures. CRC, Colorectal Cancer

The serum levels of chitinase were analyzed in 386 patients with CRC, including Group 1 and Group 2, at their first visit to determine whether chitinase was overexpressed in patients with CRC compared with healthy controls. Additionally, sera from Group 3 were tested as a control. Demographics and characteristics of all patients with CRC and healthy controls are summarized in Additional file 1: Table S1. The ELISA results revealed that serum chitinase activity was significantly higher in patients with CRC than in healthy controls [median (interquartile range, IQR) (ng/μL): 21.13 (17.35–26.16) vs 17.21 (15.39–21.27); *P* < 0.0001, Mann–Whitney test] (Fig. 1). Based on the cutoff value (23.162, *P* = 0.0092) of chitinase activity for OS from the ROC curve (Additional file 2: Figure S1), patients with CRC were divided into the high chitinase activity group (Median OS time, 32.467 months) and the low chitinase activity group (Median OS time, 39.367 months). The Kaplan–Meier survival analysis indicated that higher chitinase activity was significantly associated with poor survival in all patients with CRC (Fig. 2a, log-rank *P* = 0.0012). The correlation between CEA level and the OS of patients with CRC was initially analyzed by univariate analysis. A high CEA level was significantly associated with poor survival in all patients with CRC (Fig. 2b, *P* = 0.0009). However, the multivariate Cox’s analysis revealed that neither chitinase activity [hazard ratio (HR), 1.069; 95% confidence interval (CI), 0.671–1.701; *P* = 0.780] nor CEA level (HR, 1.276; 95% CI; 0.788–2.064; *P* = 0.322) was an independent prognostic factor of OS in patients with CRC (Additional file 1: Table S2). However, N stage (HR, 1.96; *P* = 0.011), M stage (HR, 5.33; *P* < 0.001), and histological type (HR, 2.15; *P* = 0.022) were identified as independent prognostic factors.

### Chitinase activity showed a significant correlation with liver metastases in patients with CRC

The serum levels of chitinase activity were analyzed in Group 1 and Group 2 to determine chitinase activity in the serum of patients with CRC at different tumor stages. The ELISA results revealed that serum chitinase activity was significantly higher in Group 2 compared with Group 1 [median IQR (ng/μL): 31.14 (23.69–38.37) vs 20.66 (16.85–24.60); *P* < 0.0001, Mann–Whitney test] (Fig. 1). Detailed demographics and characteristics of the two groups are summarized in Additional file 1: Table S3. Chitinase activity in Group 4 was also analyzed to confirm whether chitinase activity was associated with metastases. Chitinase activity was significantly higher in Group 2 than Group 4 [median IQR (ng/μL): 31.14 (23.69–38.37) vs 20.53 (17.38–23.98), *P* < 0.0001, Mann–Whitney test] (Additional file 2: Figure S2A). Moreover, the CEA level was significantly higher in Group 2 than Group 1 (median IQR (ng/mL): 22.50 (8.37–70.09) vs 3.71 (2.13–8.69), *P* < 0.0001, Mann–Whitney test] (Additional file 2: Figure S2B). Interestingly, multiple liver metastases had significantly higher chitinase activity compared with single liver metastasis in patients with CRC (*P* = 0.0223, Mann–Whitney test) (Additional file 2: Figure S3A). Demographics and characteristics of CRC patients with single and multi-synchronous liver metastases were summarized in Additional file 1: Table S4. Chitinase activity was measured before radical surgery in patients with CRC. Chitinase activity was upregulated in Group 1A compared with Group 1B [median IQR (ng/μL): 23.3846 (18.4999–27.4225) vs 20.1088 (16.5437–23.8659); *P* = 0.0005, Mann–Whitney test], indicating a correlation between previous chitinase activity and metachronous metastases during follow-up **(**Fig. 1). Further subanalysis with a stage-by-stage comparison showed that at both early stage [stages I and II; median IQR (ng/μL): 25.95 (22.10–29.68) vs 20.48 (17.10–23.39); *P* = 0.0038, Mann–Whitney test] and advanced stage [stage III; median IQR (ng/μL): 23.28 (18.32–26.83) vs 19.77 (15.46–24.21); *P* = 0.0156, Mann–Whitney test], Group 1A had higher chitinase activity compared with Group 1B (Additional file 2: Figure S3B).

The correlations between chitinase activity and various clinicopathological variables were investigated in all patients with CRC to further identify the clinical significance of chitinase activity in patients with CRC (Additional file 1: Table S5). Chitinase activity was significantly associated with sex (*P* = 0.031), age (*P* = 0.002), distant metastasis (*P* < 0.0001), and TNM stage (*P* < 0.0001). The ROC analysis revealed that the area under the curve (AUC) of chitinase was 0.815, with a corresponding sensitivity of 70.59% and a specificity of 80.30% in discriminating patients with or without metastasis (Additional file 2: Figure S4A). Univariate and multivariate Cox’s analyses confirmed (Additional file 1: Table S6) chitinase activity as an independent marker in discriminating Group 2 from Group 1 (univariate analysis: HR, 6.594; 95% CI, 3.611–12.043; *P* < 0.0001; multivariate analysis: HR, 3.240; 95% CI, 1.118–6.078; *P* = 0.001).

### Chitinase activity, but not CEA level, is an independent biomarker that could prognosticate liver metastases in CRC in the training and testing sets

Since chitinase activity was significantly upregulated and exhibited metastasis-discriminating power in the serum of Group 2 compared with Group 1, we next wanted to determine whether chitinase activity, as a biomarker, could prognosticate metastasis in patients with CRC at pathological stages I–III. Demographics and characteristics of patients with CRC after radical resection are summarized in Additional file 1: Table S7. Random process divided Group 1 into the training and testing sets. Clinical characteristics of patients according to chitinase activity in the training and testing sets are summarized in Additional file 1: Table S8. There was no significant association between chitinase activity and most clinical characteristics in any set. Patients with CRC in the training set were then divided into high and low chitinase activity groups based on the ROC analysis (cutoff value = 21.4154), revealing that the AUC was 0.71, with a corresponding sensitivity of 75% and a specificity of 71.26% (*P* = 0.0166) for prognosticating metastases during follow-up (Additional file 2: Figure S4B). These results indicate that chitinase activity can prognosticate metastasis in patients with CRC at stages I–III. Meanwhile, CEA was not significantly elevated in Group 1A compared with Group 1B (Additional file 2: Figure S2C) [median IQR (ng/mL): 4.51 (2.76–9.28) vs 3.55 (1.99–6.75); *P* = 0.083, Mann–Whitney test]. Next, the Kaplan–Meier test was used to analyze the metastatic rates in the high and low chitinase activity groups in the training and testing sets. The Kaplan–Meier curve showed that either in the training set (Additional file 2: Figure S5A, *P* = 0.0032) or in the testing set (Additional file 2: Figure S5B, *P* = 0.0284), patients with high chitinase activity had a significantly higher risk of developing metastases compared with patients with low chitinase activity at stages I–III. Consistent results were obtained in the combined patient population (Fig. 2c) (*P* = 0.0007). Correspondingly, a subanalysis with a stage-by-stage comparison for these two groups was performed to analyze the metastatic rate. At both early stage (Additional file 2: Figure S5C; stages I and II; log-rank test, *P* = 0.0029) and advanced stage (Additional file 2: Figure S5D; stage III; log-rank test, *P* = 0.0264), patients with high chitinase activity were more likely to metastasize compared with patients with low chitinase activity. A paralleled analysis was also performed to investigate the role of CEA in prognosticating the occurrence of metastasis in CRC considering the significant diffidence in the CEA level between Group 2 and Group 1. However, the Kaplan–Meier analysis revealed no significant difference in metastasis occurrence between patients with high and low CEA levels at stages I–III (Fig. 2d; log-rank test, *P* = 0.4054). The subanalysis of the Kaplan–Meier test with a stage-by-stage comparison showed that patients with a high CEA level had the same occurrence of metastases as patients with a low CEA level at either early stage (Additional file 2: Figure S5E; stages I and II; log-rank test, *P* = 0.1177) or advanced stage (Additional file 2: Figure S5F; stage III; log-rank test, *P* = 0.5523).

**Fig. 3:**
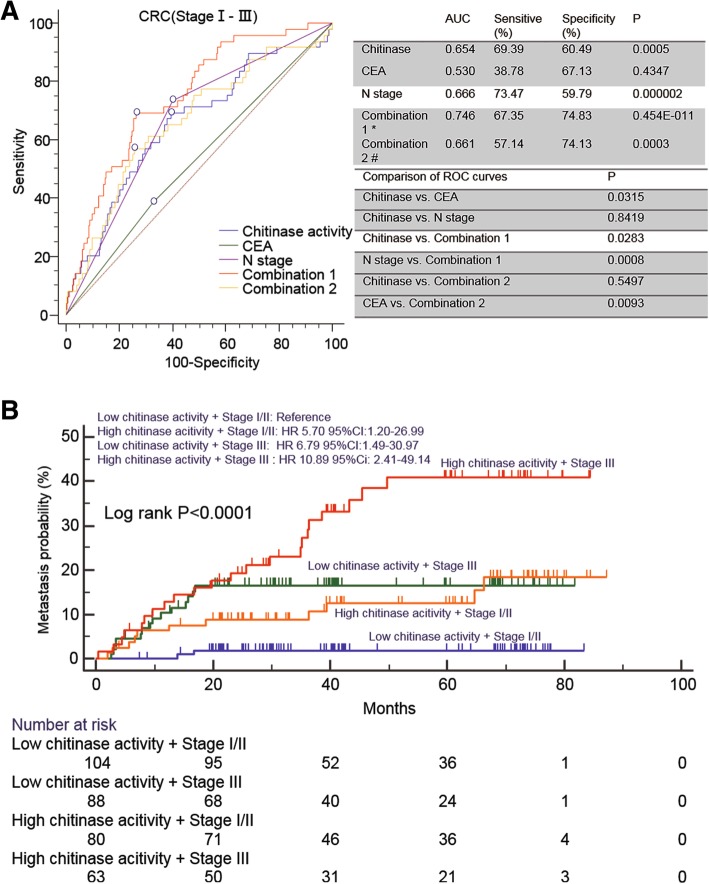
Chitinase activity combined with N stage but not CEA level prognosticates metastasis in CRC patients. **a** Receiver Operating Characteristic (ROC) analysis showed that chitinase activity combined with N stage but not CEA played an effective role in metastasis prognosis for CRC patients with Stage І-III* Combination 1 denotes that chitinase activity and N stage. # Combination 2 denotes that chitinase activity and CEA level. Equation: Combination 1 = 0.066*chitinase activity+ 1.301*N stage (N0, 0; N1/N2, 1). Combination 2 = 0.2322*chitinase activity+ 0.06718*CEA (CEA < 5, 0; CEA ≥ 5, 1) Equation coefficients were obtained from multivariate Cox’s analysis. **b** Kaplan–Meier curves of metastasis probability of CRC patients subgrouped by chitinase activity and TNM. Hazard ratios and 95% CIs were calculated by the multi-variate Cox proportional hazards regression model, adjusted for age, sex, tumor location, histological type, tumor size, gross tumor type, schistosomiasis history and treatment methods as covariates

The univariate Cox’s proportional hazards analysis in Group 1 (Table 1) revealed that only high chitinase activity and N stage were associated with the occurrence of metastasis among the training, testing and total sets. Next, the variables of chitinase activity and N stage were included in the multivariate Cox’s analysis in the training, testing and total sets. The multivariate analysis showed that chitinase activity and N stage played a significant role as independent factors in prognosticating metastasis in all sets. The subanalysis of Cox’s analysis with a stage-by-stage comparison (Table 2) confirmed that chitinase activity was an independent factor in prognosticating metastasis at both early stage (stages I and II; HR, 7.101, *P* = 0.010) and advanced stage (stage III; HR, 2.100, *P* = 0.03). Since the multivariate analysis revealed that N stage and chitinase activity had the power to prognosticate the occurrence of metastasis in CRC with stage І- III, an equation was obtained based on the multivariate Cox’s analysis (0.066 × chitinase activity + 1.301 × N stage; N0 = 0; N1/N2 = 1). For comparison, chitinase activity and CEA level were also combined to obtain an equation based on the multivariate Cox’s analysis (0.06718 × chitinase activity + 0.2322 × CEA; CEA < 5, 0; CEA ≥ 5, 1). The ROC curve analysis (Fig. 3a) based on metastasis until the last follow-up confirmed that the combination of chitinase activity and N stage significantly increased the prognostic power (AUC = 0.746; sensitivity = 67.35%; specificity = 74.83%; *P* = 0.454E-011), which was superior to either chitinase activity (AUC = 0.654; sensitivity = 69.39%; specificity = 60.49%; *P* = 0.511E-003) or N stage (AUC = 0.666; sensitivity = 73.47%; specificity = 59.79%; *P* = 0.204E-005) alone. The pairwise comparison of the ROC curve analysis revealed that the combination curve was significantly different from chitinase activity (*P* = 0.0283) and N stage (*P* = 0.008) curves, but the chitinase activity curve was not significantly different from the N stage curve (*P* = 0.8419). However, the ROC curve of CEA (Fig. 3a) showed that it had no prognostic power for metastasis in CRC (AUC = 0.530; sensitivity = 38.78%; specificity = 67.13%; *P* = 0.4347). The pairwise comparison of the ROC curve analysis revealed that the combination curve was not significantly different from the chitinase activity curve (*P* = 0.5497), indicating no significant improvement in the power of prognosticating the occurrence of metastasis after chitinase activity was combined with CEA. These data suggest that chitinase activity, but not CEA, is an independent biomarker that can prognosticate the occurrence of liver metastases in CRC. Moreover, the combination with N stage, but not CEA, improved the prognostic power for metastasis in CRC. Considering the metastasis risk heterogeneity in the same TNM stage, we evaluated the prognostication value of chitinase activity in different TNM stages in the combined patient population. Kaplan–Meier analyses (Fig. 3b) showed that patients with high chitinase activity at TNM stage III had the highest metastasis risk, whereas those with low chitinase activity at TNM stage I/II had the lowest metastasis risk (*P* < 0.0001). Interestingly, patients with high chitinase activity at TNM stage I/II had a similar metastasis risk compared with those with low chitinase activity at TNM stage III, indicating that patients with high chitinase activity at TNM stage I/II need further intervention post-operation.

**Table 1:** Univariate and Multivariable Cox regression analysis of CRC patients’ metastasis probability in training, testing and total sets for identifying the prognosis factors for metastasis between metachronous metastasis CRC patients and non-met CRC patients after last follow-up

Characteristics	Training Set		Testing Set		Total Set	
	HR(95% CI)	*p* Value	HR(95% CI)	*p* Value	HR(95% CI)	*p* Value
*All CRC patients with Stage I – III at first visit (Univariate Cox proportional hazards models)*						
Age (>median vs. ≤ median)	0.786 (0.249–2.477)	0.680	1.671 (0.851–3.282)	0.136	1.395 (0.789–2.466)	0.251
Sex (male vs. female)	1.776 (0.535–5.889)	0.348	1.181 (0.612–2.278)	**0.019**	1.313 (0.739–2.334)	0.351
T stage (T3/T4 vs. T1/T2)	3.125 (0.403–24.251)	0.276	2.216 (0.680–7.215)	0.187	2.474 (0.890–6.879)	0.073
N stage (N1/N2 vs. N0)	6.677 (1.462–30.493)	**0.014**	3.153 (1.557–6.385)	**0.001**	3.686 (1.954–6.951)	**0.000**
CEA (≥5 vs. < 5)	1.560 (0.503–4.838)	0.442	1.158 (0.589–2.275)	0.67	1.276 (0.718–2.267)	0.405
Tumor location (Colon vs. Rectum)	1.006 (0.324–3.128)	0.992	1.217 (0.635–2.334)	0.554	1.165 (0.663–2.046)	0.596
Histological type (Mucus adenocarcinoma vs. Adenocarcinoma)	2.609 (0.571–11.923)	0.216	1.55 (0.546–4.397)	0.41	1.812 (0.769–4.274)	0.168
Tumor size (≥Median vs. <Median)	1.089 (0.345–3.436)	0.884	1.103 (0.578–2.107)	0.766	1.084 (0.617–1.904)	0.778
Schistosomiasis history (Yes vs. No)	1.991 (0.254–15.608)	0.512	0.920 (0.221–3.826)	0.909	1.131 (0.352–3.638)	0.836
Treatment (Surgery vs. Surgery+ Postoperative chemotherapy)	3.588 (0.786–16.375)	0.099	2.334 (1.025–5.313)	0.044	2.562 (1.241–5.290)	**0.011**
Chitinase enzyme activity (≥Cut-off vs. <Cut-off)	5.670 (1.535–20.948)	**0.009**	2.094 (1.065–4.115)	**0.032**	2.703 (1.487–4.912)	**0.001**
All CRC patients with Stage I – III at first visit (Multivariate Cox proportional hazards models) *						
N stage (N1/N2 vs. N0)	6.413 (1.403–29.311)	**0.017**	3.229 (1.594–6.54)	**0.001**	3.726 (1.976–7.027)	**0.000**
Chitinase enzyme activity (≥Cut-off vs. <Cut-off)	5.443 (1.472–20.122)	**0.011**	2.168 (1.103–4.261)	**0.025**	2.739 (1.507–4.978)	**0.001**

Bold values indicate statistically significant

**Table 2:** Subanalysis of stage-by-stage of Cox proportional hazards models analysis for identifying the prognosis factors for metastasis between metachronous metastasis CRC patients and non-met CRC patients after last follow-up

Characteristics	Univariate			Multivariate		
	HR	95% CI	*p* Value	HR	95% CI	*p* Value
*All CRC patients with Stage I – III at first visit (Cox proportional hazards models)*						
Age (≥median vs. <median) median = 62 y	1.395	0.789–2.466	0.251			
Sex (male vs. female)	1.313	0.739–2.334	0.351			
T stage (T3/T4 vs. T1/T2)	2.474	0.890–6.879	0.073			
N stage (N1/N2 vs. N0)	3.686	1.954–6.951	**< 0.0001**	3.133	1.590–6.174	**0.001**
CEA (≥5 vs. < 5)	1.276	0.718–2.267	0.405			
Tumor location (Colon vs. Rectum)	1.165	0.663–2.046	0.596			
Histological type (Mucus adenocarcinoma vs. Adenocarcinoma)	1.812	0.769–4.274	0.168			
Tumor size (≥Median vs. <Median)	1.084	0.617–1.904	0.778			
Schistosomiasis history (Yes vs. No)	1.131	0.352–3.638	0.836			
Treatment (Surgery vs. Surgery+ Postoperative chemotherapy)	2.562	1.241–5.290	**0.011**	0.605	0.279–1.312	0.203
Chitinase enzyme activity (≥Cut-off vs. <Cut-off)	2.703	1.487–4.912	**0.001**	2.785	1.532–5.064	**0.001**
*CRC patients with Stage I and II at first visit (Cox proportional hazards models)* ^*a*^						
Age (≥median vs. <median) Median = 63 y	2.157	0.704–6.609	0.168			
Sex (male vs. female)	0.952	0.320–2.833	0.930			
T stage (T3/T4 vs. T1/T2)	0.811	0.250–2.636	0.727			
CEA (≥5 vs. < 5)	2.334	0.782–6.968	0.118			
Tumor location (Colon vs. Rectum)	1.115	0.374–3.320	0.845			
Tumor size (≥Median vs. <Median)	1.058	0.356–3.149	0.919			
Schistosomiasis history (Yes vs. No)	1.151	0.150–8.857	0.892			
Treatment (Surgery vs. Surgery+ Postoperative chemotherapy)	0.371	0.114–1.206	0.086			
Chitinase enzyme activity (≥Cut-off vs. <Cut-off)	7.101	1.585–31.814	**0.010**	7.101	1.585–31.814	**0.010**
*CRC patients with Stage III at first visit (Cox proportional hazards models)*						
Age (≥median vs. <median) median = 63 y	1.219	0.631–2.352	0.556			
Sex (male vs. female)	1.316	0.666–2.600	0.429			
CEA (≥5 vs. < 5)	0.962	0.492–1.881	0.910			
Tumor location (Colon vs. Rectum)	1.119	0.579–2.164	0.738			
Histological type (Mucus adenocarcinoma vs. Adenocarcinoma)	1.518	0.630–3.655	0.352			
Tumor size (≥Median vs. <Median)	1.071	0.554–2.069	0.838			
Schistosomiasis history (Yes vs. No)	1.453	0.349–6.054	0.608			
Treatment (Surgery vs. Surgery+ Postoperative chemotherapy)	0.845	0.328–2.179	0.727			
Chitinase enzyme activity (≥Cut-off vs. <Cut-off)	2.100	1.078–4.093	**0.03**	2.100	1.078–4.093	**0.03**

Bold values indicate statistically significant

^a^N stage is excluded for patients with Stage I and II are at N0 stage

### Chitinase promoted the migration of CRC cells in vitro

**Fig. 4:**
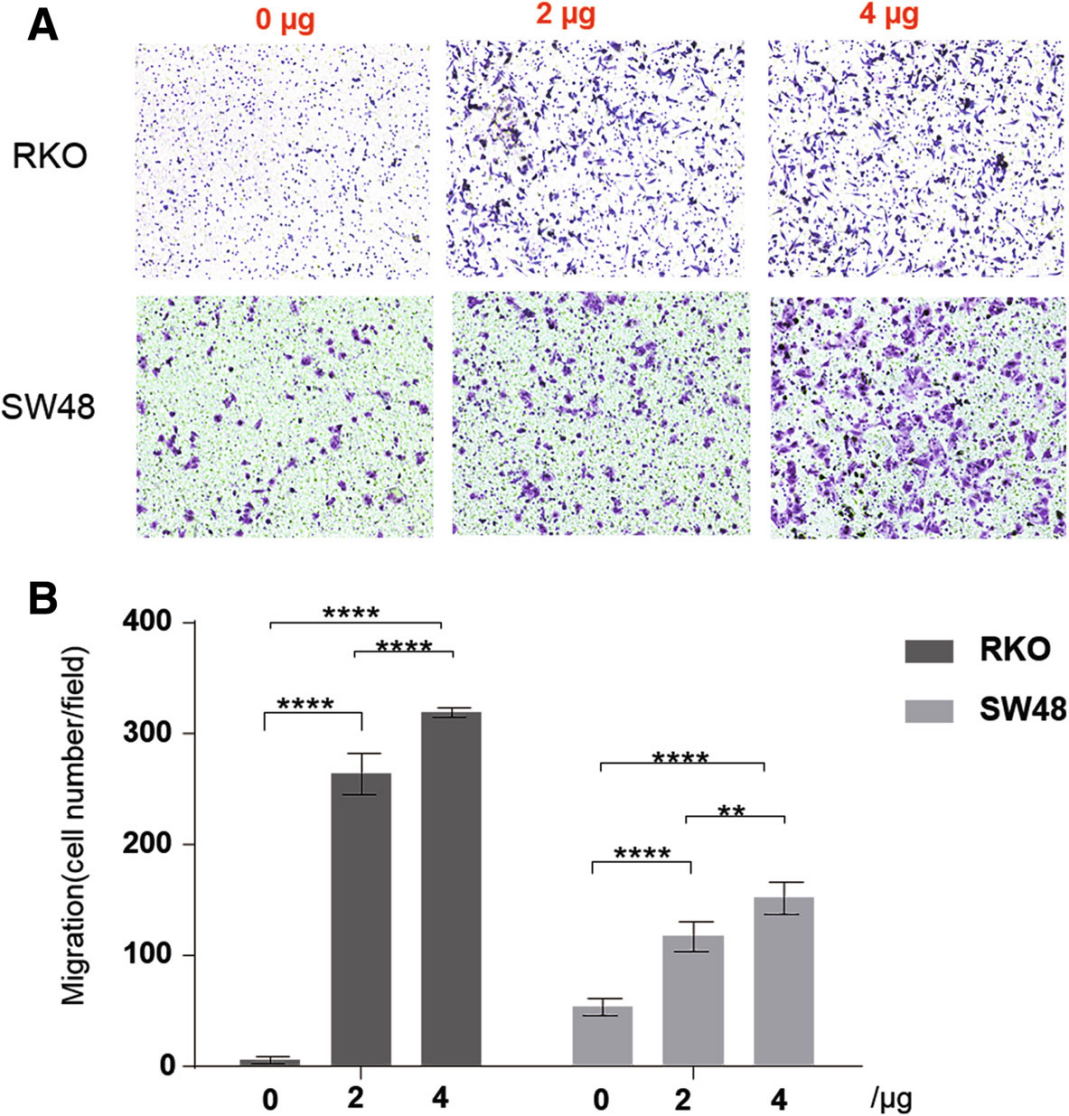
Chitinase promotes migration of colorectal cancer cells in vitro. (A) RKO cells and SW48 cells were treated with chitinase by 0, 2 μg and 4 μg, respectively. Images shown were representative at each concentration in each cell line. (B) Histogram of RKO cell line is in Black and SW48 cell line is in Gray. Each bar with its error bar represented Mean ± standard deviation (SD) at one concentration. **P < 0.01, ****P < 0.0001, one-way analysis of variance with Tukey’s multiple comparisons test. All tests were at two sides

To further investigate whether chitinase plays a direct role in cancer metastasis, a Transwell migration assay was performed using RKO and SW48 cell lines. Both RKO and SW48 cell lines were treated with 0 μg, 2 μg, or 4 μg chitinase protein. The number of RKO and SW48 cells (Fig. 4a) through the Transwell significantly increased with the increase in chitinase concentration: the number of RKO and SW48 cells at 4 μg (mean, 318.25/field and 151.5/field, respectively) significantly increased compared with that at 2 μg (mean, 263.5/field, *P* < 0.0001, and 116.75/field, *P* < 0.0001, respectively) and at 0 μg (mean 6.25/field, *P* < 0.0001, and 53.5/field, *P* < 0.01, respectively) (Fig. 4b, ANOVA with Tukey’s multiple comparison test). Moreover, the cell lines were treated with 10 μg chitinase, which resulted in rapid cell death (data not shown). Taken together, these data indicate that chitinase promotes CRC cell migration in vitro.

## Discussion

This study demonstrated the potential role of serum chitinase activity as a noninvasive biomarker for CRC metastases. It highlighted the clinical significance of serum chitinase activity for determining patient prognosis and CRC metastases. It also demonstrated that preoperative serum chitinase activity is better than serum CEA for prognosticating metastases in CRC. Moreover, the results were substantiated through in vitro experiments.

Nonanatomic cancer prognostic factors are important for the risk stratification of patients with CRC and the choice for further treatment in patients with early-stage CRC after surgery [10]. However, no recognized biomarkers with sufficient data are available at present to determine the prognosis of patients with CRC [10]. Currently, it is believed that tumor stage, tumor differentiation, and DNA mismatch status are important prognostic factors for deciding further treatment strategies [11]. However, these biomarkers are difficult for general use because of unavoidable technical difficulties inherent in the subjective definition and discriminant analysis [10]. Moreover, gene signatures specific for intestinal stem cells have broad implications but cannot be feasibly translated into clinical applications due to certain limitations [12].

CEA is commonly used to monitor cancer relapse after curative resection. Further evaluation by diagnostic imaging is recommended if the blood CEA level increases above threshold. However, false-positive results can be caused by smoking and certain diseases. The univariate analysis in this study showed that both preoperative CEA level and chitinase activity were significantly correlated with metastases and prognosis; however, the multivariate analysis showed that chitinase activity was superior to CEA in predicting metastases in CRC.

The 18 Chitinases and human chitinase-like proteins (CLPs) secreted by macrophages, neutrophils, and cancer cells are most likely to influence tumor progression, as the majority of these cells are located in the tumor microenvironment [8, 13]. Moreover, chitinase and human CLPs play important roles in inflammation, tissue remodeling, and tissue injury [13]. Previous studies have described an association between genes of the 18-glycosyl hydrolase family of chitinases and certain types of cancer [14, 15]. Recent evidence showed that inflammation can promote cancer development and metastases. The chitinase family could induce the generation of pro- and anti-inflammatory cytokines and chemokines, such as interleukin (IL)-1β, IL-6, IL-12, and IL-13, making them potential modulators in an inflammatory tumor microenvironment [8, 16]. Inflammation plays a key role in cancer progression [17]. Therefore, the correlations between chitinase and cancer and inflammation require further investigation. Tumor-associated macrophages in breast cancer as major innate immune cells secrets CLPs which could regulate intra-tumoral immunity and angiogenesis [18]. Furthermore, high CLPs expression in tumors after neoadjuvant chemotherapy was associated with increased risk of distant metastasis in breast cancer [18]. A study using ELISA assay to detect the concentration of chitinase 3-like 1 in the serum indicated that higher chitinase 3-like 1secreted by peri-tumoral macrophages in esophageal carcinoma had a significantly poor overall survival [19]. More than macrophages, chitinase is also related to epithelial-mesenchymal transition (EMT). Jefri and his colleague in their study proposed that YKL-40, one member of chitinase family, regulated EMT and migration/invasion enhancement by detecting the EMT markers such as Twist, N-cadherin, Vimentin, and E-cadherin in non-small cell lung cancer [20]. Also they found that elevated YKL-40 expression was correlate with the phenotypic characteristics of metastasis in lung cancer [20]. These studies above indicated that macrophage secretion in humans is one main source of chitinase and elevated expression of chitinase is correlate with the occurrence of metastasis in cancer.

However, no studies have reported on the association of chitinase activity in serum with the metastasis of CRC. Chitinase enzyme is extremely stable [21], suggesting that the enzyme activity assay evaluating metastases in CRC is feasible and not influenced by other factors. Moreover, the enzyme activity assay has some advantages including measuring a biological function with high specificity and reliability and avoiding cross-contamination by unspecific protein binding, which is prior to the measurement of the protein.

This study provided experimental proof that chitinase activity in serum represents a novel, noninvasive biomarker for prognosticating metastasis in CRC. A significant difference in chitinase activity was observed in the serum of patients with CRC relative to healthy controls. Then, chitinase activity was tested in patients with CRC with or without metastasis. Chitinase activity was revealed as an independent biomarker with the power to distinguish patients with CRC having synchronous liver metastasis from patients with CRC without metastasis at their first visit after analyzing together with age, sex, T stage, N stage, CEA level, tumor location, tumor size, histological type, and schistosomiasis history in the multivariate Cox’s analysis. Therefore, the present study focused on patients with CRC without metastasis at their first visit with a long-term follow-up, investigating whether chitinase activity could prognosticate the occurrence of metastasis in patients with early-stage CRC. Interestingly, chitinase activity, but not CEA level, in patients with CRC having metachronous metastasis was significantly higher compared with patients with CRC without metastasis until the last follow-up both in the training and testing sets. Furthermore, the multivariate Cox’s analysis, which included age, sex, T stage, N stage, CEA level, tumor location, tumor size, histological type, therapeutic method, schistosomiasis history, and chitinase activity (dichotomized by the ROC curve), revealed that chitinase activity, but not CEA level, was an independent prognosis of metastasis in patients with CRC. Of note, a subanalysis that used Cox’s analysis models by the American Joint Committee on Cancer TNM Stage revealed that patients with CRC having high chitinase activity, whether at early stage (stages I and II) or advanced stage (stage III), were at high risk of metastasis (either in the training or testing set), indicating that essential intervention measures must be taken for these high-risk patients. Moreover, the ROC analysis revealed that the combination of chitinase activity with N stage, but not with CEA, had the best power of prognosticating the occurrence of metastasis in patients with CRC. Interestingly, high chitinase activity at TNM stage I/II had the same metastasis risk with low chitinase activity at TNM stage III, indicating that patients with high chitinase activity at TNM stage I/II need further intervention post-operation. More multi-centers prospective studies are required to further clarify the role of chitinase in evaluating the metastasis risk of patients with early-stage CRC.

Patients with CRC stage II showed good prognosis with a low occurrence of metastases and a 5-year survival rate of 72–85% [22]. Only patients with proficient mismatch repair tumors and clinicopathological high-risk features, particularly those with T4 cancers or multiple high-risk features such as obstruction, perforation, and poor differentiation, are treated with chemotherapy in clinical practice. However, the present study showed that high chitinase activity is associated with a high risk of metastases. These data indicate that patients with high chitinase activity might benefit from adjuvant chemotherapy and strict supervision. Therefore, further clinical trials should be performed in patients with CRC stage II to decrease the chances of distant metastases and improve patient survival.

In addition to clinical data, a Transwell migration assay was also performed to substantiate these results. RKO and SW48 CRC cell lines treated with chitinase showed significant cancer cell migration, indicating that chitinase activity promoted cancer metastases. Studies on inhibiting chitinase activity in the liver metastasis model with high chitinase activity should be performed. Whether cells or tumor tissues secrete chitinase also needs to be explored. Moreover, it is still unknown whether upregulating chitinase expression in the CRC model can induce metastases in vivo.

There are some limitations to this study. Although a sample size of 386 is not small, dividing subjects into groups with or without metastasis and into training and validation sets may have made each group small. We will continue to conduct ongoing follow-up studies and increase the sample size to obtain a higher level of evidence.

## Conclusions

Taken together, this novel study demonstrated the clinical significance of chitinase activity in the blood as a nonanatomic cancer biomarker for prognosticating the occurrence of metastasis in patients with CRC. Moreover, the combination of chitinase activity and N stage had the best power of prognosticating the occurrence of metastasis in patients with CRC. Considering the exploratory design of this study, randomized clinical trials are needed to confirm these findings, which is a prerequisite for designing further interventions for patients with CRC at high risk of metastasis.

## Additional files

Additional file 1: Supplementary Tables. (DOCX 54 kb)

Additional file 2: Supplementary Figures. (PPTX 191 kb)

## Data Availability

All data generated or analyzed during this study are included in this published article. Further details are available from the corresponding author upon request.

## Abbreviations

AUC: Area under the curve
CEA: Carcinoembryonic antigen
CHI3L1: Chitinase-3-like protein 1
CI: Confidence interval
CLP: Chitinase-like proteins
CRC: Colorectal cancer
DMEM: Dulbecco’s modified Eagle’s Medium
ELISA: Enzyme-linked immunosorbent assay
FBS: Fetal bovine serum
HR: Hazard ratio
IQR: Interquartile range
OS: Overall survival
RFS: Recurrence free survival
ROC: Receiver operating characteristic
SRRSH: Sir Run Run Shaw Hospital
