# Supplementary material for: Serum chitinase activity prognosticates metastasis of colorectal cancer
Source: BMC Cancer. 2019 Jun 25;19:629. doi: 10.1186/s12885-019-5834-7 (PMC6593502; doi:10.1186/s12885-019-5834-7)
Supplement: Supplementary file 1 — Supplementary Tables. (DOCX 54 kb) [file 12885_2019_5834_MOESM1_ESM.docx]

***Serum Chitinase Activity Prognosticates Metastasis Of Colorectal Cancer***

**Supplement Table**

| Table S1: Demographics and characteristics of all patients with colorectal cancer and healthy controls | | | |
| --- | --- | --- | --- |
| Characteristics | CRC (n=386) | Healthy controls(n=100) | P value |
| Age, y, median (IQR)  Mean(SD) | 62(54-71)  62(12) | 61(51-67)  60(10) | 0.078* |
| Sex  Male, No. (%)  Female, No. (%) | 222(57.5)  164(42.5) | 58(58)  42(42) | 0.93^#^ |
| Chitinase activity, median (IQR) | 21.1357(17.3510-26.1626) | 17.2142(15.3977-21.2735) | **<0.0001**** |
| * By T test.  # By Chi-squared test.  **By Mann-Whitney test.  Bold values indicate statistically significant. | | | |

| Table S2: Cox proportional hazards models for survival prognosis between synchronous liver-met CRC patients and non-met CRC patients at first visit. | | | | | | | |
| --- | --- | --- | --- | --- | --- | --- | --- |
| Characteristics | HR | Univariate  95% CI | p Value | HR | Multivariate  95% CI | P Value | |
| Age(≥median vs. <median) | 1.429 | 0.942-2.168 | 0.093 |  |  | |  |
| Sex(male vs. female) | 1.169 | 0.770-1.774 | 0.463 |  |  | |  |
| T stage(T3/T4 vs. T1/T2) | 2.991 | 1.209-7.402 | **0.018** | 1.788 | 0.699-4.521 | | 0.227 |
| N stage(N1/N2 vs. N0) | 2.929 | 1.800-4.766 | **<0.0001** | 1.965 | 1.169-3.304 | | **0.011** |
| M stage(M1 vs. M0) | 10.828 | 6.855-17.106 | **<0.0001** | 5.313 | 2.839-9.941 | | **<0.0001** |
| CEA(≥5 vs. <5) | 2.010 | 1.333-3.029 | **0.001** | 1.276 | 0.788-2.064 | | 0.322 |
| Tumor location(Colon vs. Rectum) | 0.873 | 0.580-1.315 | 0.517 |  |  | |  |
| Histological type(Mucus adenocarcinoma vs. Adenocarcinoma) | 2.368 | 1.247-4.497 | **0.008** | 2.150 | 1.116-4.141 | | **0.022** |
| Tumor size(≥Median vs. <Median) | 1.180 | 0.753-1.849 | 0.470 |  |  | |  |
| Schistosomiasis history(Yes vs. No) | 1.696 | 0.780-3.688 | 0.183 |  |  | |  |
| Chitinase enzyme activity (≥Cut-off vs. <Cut-off) | 1.982 | 1.315-2.985 | **0.001** | 1.069 | 0.671-1.701 | | 0.780 |
| Bold values indicate statistically significant. | | | | | | | |

| Table S3: Demographics and characteristics of all patients with colorectal cancer* | | | |
| --- | --- | --- | --- |
| Characteristics | CRC with synchronous  liver metastasis (n=51) | CRC without metastasis at first-visit (n=335) | P value |
| Age, y, median (IQR)  Mean(SD) | 63(53-69)  60.73(12.01) | 62(55-72)  62.3(12.09) | 0.388** |
| Sex  Male, No. (%)  Female, No. (%) | 34(66.7)  17(33.3) | 188(56.1)  147(43.9) | 0.173*** |
| Chitinase, median (IQR) | 31.1424(23.6930-38.3749) | 20.6664(16.8509-24.6050) | **<0.0001^#^** |
| CEA, median (IQR) | 22.50(8.37-70.09) | 3.71(2.13-8.69) | **<0.0001^#^** |
| T stage  T1/T2, No. (%)  T3/T4, No. (%)  Tx, No. (%) | 0(0)  32(62.7)  19(37.3) | 57(17.0)  278(83.0)  / | / |
| N stage  N0, No. (%)  N1/N2, No. (%)  Nx, No. (%) | 7(13.7)  25(49.0)  19(37.3) | 184(54.9)  151(45.1)  / | / |
| Therapy method  Surgery  Surgery + postoperative chemotherapy  Chemotherapy or radiotherapy | 4(7.8)  28(54.9)  19(37.3) | 212(63.3)  123(36.7)  / | / |
| *All patients with colorectal cancer were received surgery except nineteen patients with synchronous liver metastasis, who received 5-fluorouracil-based chemotherapy or radiotherapy only.  ** By T test.  ***By Chi-squared test.  # By Mann-Whitney test.  Bold values indicate statistically significant. | | | |

| Table S4: Demographics and characteristics of CRC patients with single and multi-synchronous liver metastases | | | |
| --- | --- | --- | --- |
| Characteristics | Single metastasis (n=30) | Multi metastasis (n=21) | P value |
| Age, y, Mean(SD) | 58.93 (11.72) | 63.29 (12.24) | 0.206* |
| Sex  Male, No. (%)  Female, No. (%) | 21 (70.0)  8 (30.0) | 13 (61.9)  8 (38.1) | 0.546^#^ |
| CEA, median (IQR) | 24.58 (13.62-68.26) | 23.95 (7.80-416.70) | 0.619** |
| * By T test.  # By Chi-squared test.  **By Mann-Whitney test. | | | |

| Table S5: Clinical characteristics of serum Chitinase enzyme activity in patients with colorectal cancer* | | | |
| --- | --- | --- | --- |
|  | N | Median(IQR) | P-value |
| Sex  Male  Female | 222  164 | 21.7378(17.9240-27.9176)  20.7785(16.8671-25.0552) | **0.031** |
| Age  <Median(62)  ≥Median(62) | 184  202 | 20.1088(16.1970-24.8120)  22.0037(18.3808-27.2886) | **0.002** |
| T stage △  T1/T2  T3/T4 | 57  310 | 20.6664(17.1059-24.7803)  20.9237(17.1109-25.1424) | 0.792 |
| N stage △  N0  N1/N2 | 191  176 | 20.8876(17.4522-24.4311)  20.9194(16.6706-25.8477) | 0.801 |
| M stage  M0  M1 | 335  51 | 20.6664(16.8521-24.5906)  31.1424(23.7688-38.2464) | **< 0.0001** |
| TNM stage  I  II  III  IV | 50  134  151  51 | 20.4496(17.4438-24.7082)  20.7785(17.1372-23.5212)  20.4267(16.2977-24.9409)  31.1424(23.7688-38.2464) | **< 0.0001** |
| Primary tumor location  Rectum  Colon | 181  205 | 21.0140(17.5976-26.7794)  21.3444(17.0975-25.8094) | 0.742 |
| Histological type △  Adenocarcinoma  Mucus adenocarcinoma | 336  31 | 20.9406(17.3331-25.1508)  18.5232(15.3964-23.9869) | 0.120 |
| Tumor size(cm) △  <Median(4.5)  ≥Median(4.5) | 172  195 | 20.4557(16.5287-24.4612)  21.0434(17.6246-25.9356) | 0.206 |
| Gross tumor type △  Ulcerative  Polypoid  Infiltrating | 229  130  8 | 20.9267(17.1306-25.1100)  20.7267(17.1029-25.5759)  20.8862(17.1969-21.2172) | 0.791 |
| Schistosomiasis history △  Yes  No | 22  345 | 23.2043(19.7160-28.6797)  20.7870(16.9077-25.0787) | 0.115 |
| Bold values indicate statistically significant.  *By Mann–Whitney test or Kruskal-Wallis test.  △ All patients with colorectal cancer were received surgery or surgery with postoperative chemotherapy except nineteen patients with synchronous liver metastasis, who received 5-fluorouracil-based chemotherapy or radiotherapy only.  TNM, tumor node metastasis. | | | |

| Table S6: Cox proportional hazards model analysis for metastasis between synchronous liver-met CRC patients and non-met CRC patients at first visit | | | | | | | |
| --- | --- | --- | --- | --- | --- | --- | --- |
| Characteristics | HR | Univariate  95% CI | p Value | HR | Multivariate  95% CI | p Value | |
| Age(≥median vs. <median) | 0.974 | 0.547-1.640 | 0.847 |  |  | |  |
| Sex(male vs. female) | 0.677 | 0.378-1.212 | 0.186 |  |  | |  |
| T stage(T3/T4 vs. T1/T2) | 26.042 | 0.410-1656.145 | 0.124 |  |  | |  |
| N stage(N1/N2 vs. N0) | 3.876 | 1.676-8.961 | **0.001** | 2.607 | 1.118-6.078 | | **0.026** |
| CEA(≥5 vs. <5) | 6.955 | 3.386-14.287 | **<0.0001** | 9.132 | 3.179-26.228 | | **0.001** |
| Tumor location(Colon vs. Rectum) | 1.261 | 0.722-2.203 | 0.413 |  |  | |  |
| Histological type(Mucus adenocarcinoma vs. Adenocarcinoma) | 1.121 | 0.342-3.681 | 0.850 |  |  | |  |
| Tumor size(≥Median vs. <Median) | 1.134 | 0.564-2.280 | 0.724 |  |  | |  |
| Schistosomiasis history(Yes vs. No) | 1.045 | 0.250-4.375 | 0.951 |  |  | |  |
| Chitinase enzyme activity  (≥Cut-off vs. <Cut-off) | 6.594 | 3.611-12.043 | **<0.0001** | 3.240 | 1.118-6.078 | | **0.001** |
| Bold values indicate statistically significant. | | | | | | | |

| Table S7: Demographics and characteristics of CRC patients with metachronous metastasis and CRC patients without metastasis after last follow-up | | | |
| --- | --- | --- | --- |
| Characteristics | CRC with metachronous metastasis (n=49) | CRC without metastasis after last follow-up (n=286) | P value |
| Age, y, median (IQR)  Mean(SD) | 63(53-72)  63.12(11.73) | 62(55-72)  62.15(12.16) | 0.605* |
| Sex  Male, No. (%)  Female, No. (%) | 30(61.2)  19(38.8) | 158(55.2)  128(44.8) | 0.534^#^ |
| Chitinase, median (IQR) | 23.3846(18.4998-27.4225) | 20.1088(16.5437-23.8659) | **0.0005**** |
| CEA, median (IQR) | 4.51(2.76-9.28) | 3.55(1.99-6.75) | 0.083** |
| T stage  T1/T2, No. (%)  T3/T4, No. (%) | 4(8.2)  45(91.8) | 53(18.5)  233(81.5) | 0.098^#^ |
| N stage  N0, No. (%)  N1/N2, No. (%) | 13(26.5)  36(73.5) | 171(59.8)  115(40.2) | **<0.001^#^** |
| Therapy method  Surgery  Surgery+postoperative chemotherapy  Chemotherapy or radiotherapy | 40(81.6)  9(18.4)  / | 172(60.1)  114(39.9)  / | **0.004^#^** |
| * By T test.  # By Chi-squared test.  **By Mann-Whitney test.  Bold values indicate statistically significant. | | | |

|  | |  | |  |  |
| --- | --- | --- | --- | --- | --- |
| Table S8: Clinical characteristics of patients according to Chitinase activity in the training and testing sets | | | | | |
|  | Training Set (N=99) | | Testing Set (N=236) | | Total Set (N= 335) |
| Characteristics | Low High *p*-value | | | Low High *p*-value | Low High *p*-value |
| Age, mean(SD) | 61.12 63.41 0.360^a^  (12.10) (11.06) | | | 60.06 65.26 **0.001^a^**  (11.95) (12.06) | 60.42 64.82 **0.001^a^**  (11.98) (11.87) |
| Sex  Male  Female | 28 24 **0.011^b^**  37 10 | | | 74 62 0.895^b^  53 47 | 102 86 0.222^b^  90 57 |
| T stage  T1/T2  T3/T4 | 13 6 1.000^b^  52 28 | | | 21 17 0.861^b^  106 92 | 34 23 0.769^b^  158 120 |
| N stage  N0  N1/N2 | 37 17 0.531^b^  28 17 | | | 67 63 0.512^b^  60 46 | 104 80 0.824^b^  88 63 |
| Primary tumor location  Rectum  Colon | 33 16 0.833^b^  32 18 | | | 61 50 0.794^b^  66 59 | 94 66 0.659^b^  98 77 |
| Histological type  Adenocarcinoma  Mucus adenocarcinoma | 61 31 0.689^b^  4 3 | | | 114 101 0.497^b^  13 8 | 175 132 0.842^b^  17 11 |
| Tumor size(cm)  <Median (4.5)  ≥Median (4.5) | 28 14 1.000^b^  37 20 | | | 64 52 0.697^b^  63 57 | 92 66 0.825^b^  100 77 |
| Gross tumor type  Ulcerative  Polypoid  Infiltrating | 37 19 0.993^b^  26 14  2 1 | | | 80 69 0.689^b^  44 39  3 1 | 117 88 0.747^b^  70 53  5 2 |
| Schistosomiasis history  Yes  No | 4 2 1.000^b^  61 32 | | | 6 8 0.421^b^  121 101 | 10 10 0.496^b^  182 133 |
| Bold values indicate statistically significant.  ^a^The P-values were calculated using an unpaired Student’s t-test.  ^b^The P-values were calculated using a Pearson chi-square test. | | | | | |
