# Supplementary material for: Serum chitinase activity prognosticates metastasis of colorectal cancer
Source: BMC Cancer. 2019 Jun 25;19:629. doi: 10.1186/s12885-019-5834-7 (PMC6593502; doi:10.1186/s12885-019-5834-7)
Supplement: Supplementary file 2 — Supplementary Figures. (PPTX 191 kb) [file 12885_2019_5834_MOESM2_ESM.pptx]

## Slide 1
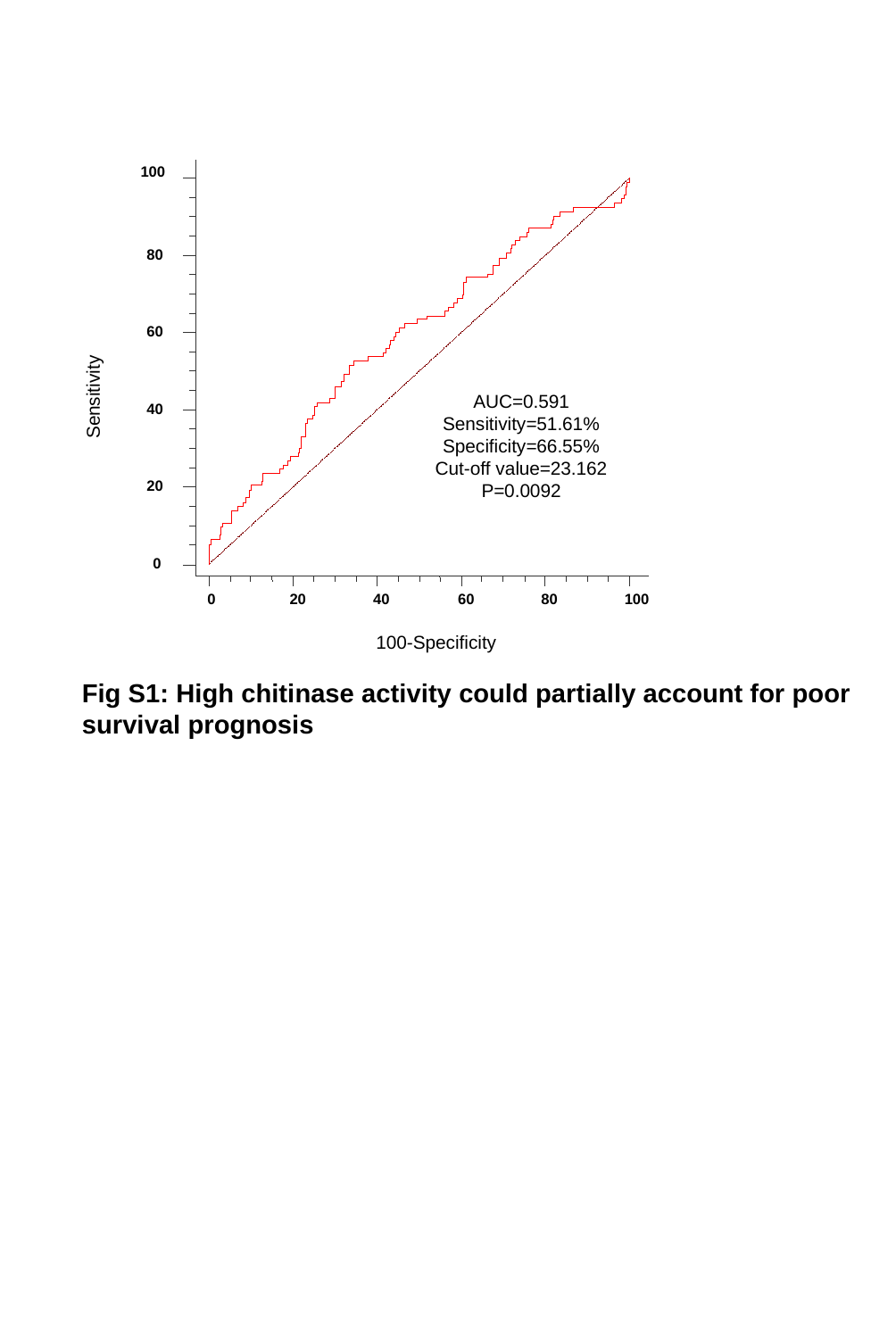

100
80
60
Sensitivity
AUC=0.591
Sensitivity=51.61%
Specificity=66.55%
Cut-off value=23.162
P=0.0092
40
20
0
0
20
40
60
80
100
100-Specificity
Fig S1: High chitinase activity could partially account for poor survival prognosis

## Slide 2
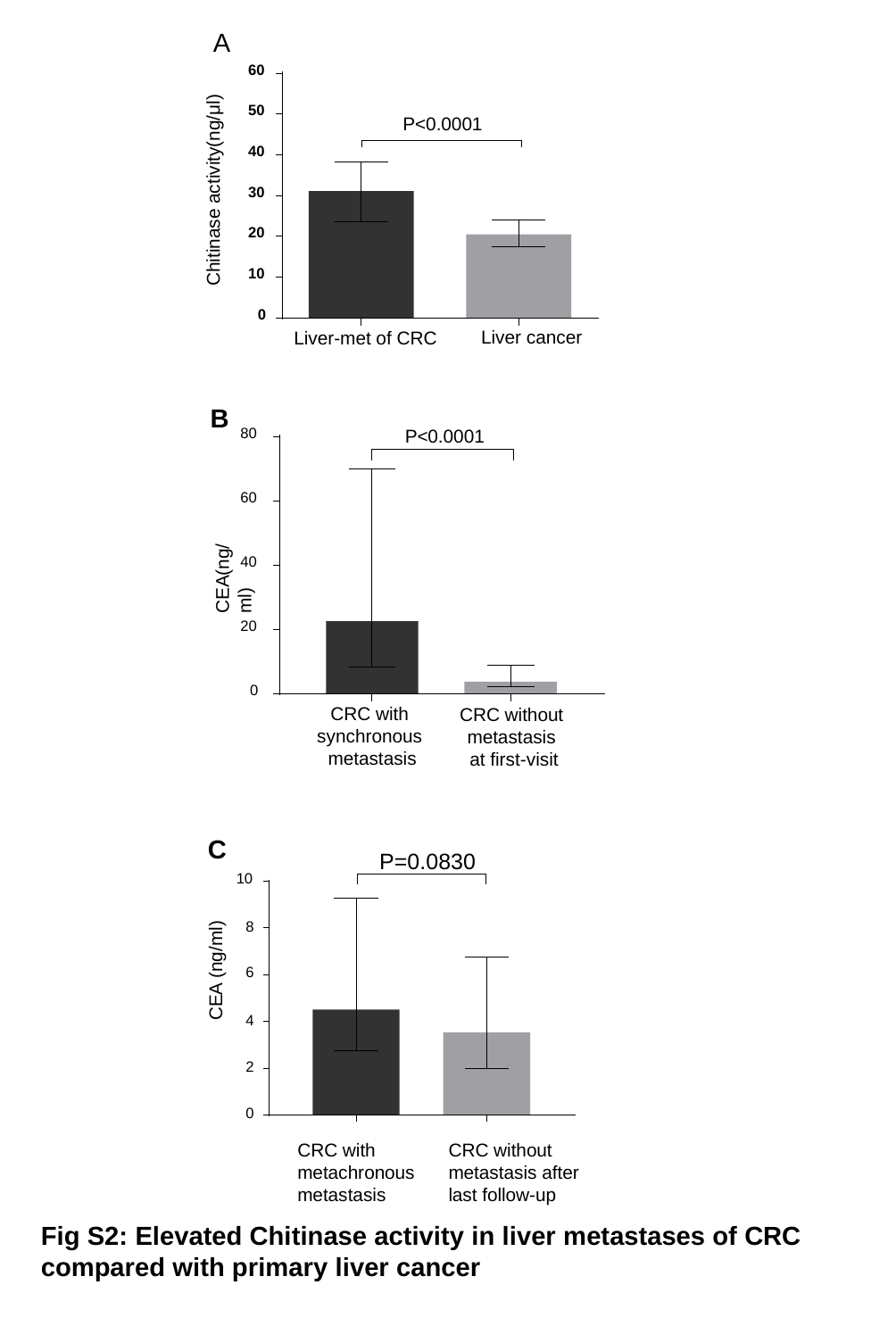

A
60
Chitinase activity(ng/μl)
50
P<0.0001
40
30
20
10
0
Liver cancer
Liver-met of CRC
B
80
P<0.0001
60
CEA(ng/ml)
40
20
0
CRC with
synchronous
metastasis
CRC without
metastasis
at first-visit
C
P=0.0830
10
8
CEA (ng/ml)
6
4
2
0
CRC with
metachronous
metastasis
CRC without
metastasis after
last follow-up
Fig S2: Elevated Chitinase activity in liver metastases of CRC compared with primary liver cancer

## Slide 3
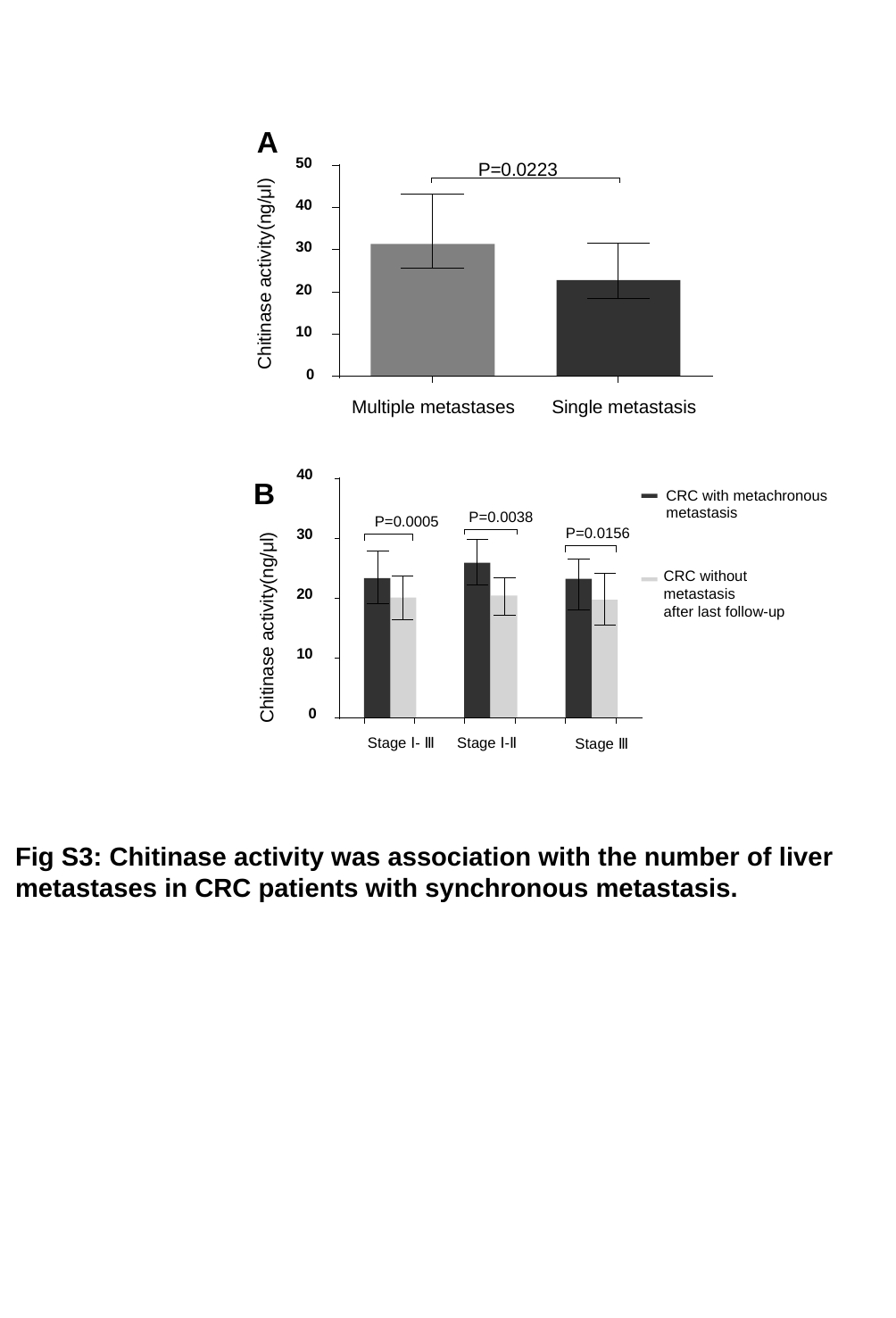

A
50
P=0.0223
40
30
20
10
0
Multiple metastases
Single metastasis
Chitinase activity(ng/μl)
Chitinase activity(ng/μl)
40
30
20
10
0
CRC with metachronous
metastasis
P=0.0038
P=0.0005
P=0.0156
CRC without metastasis
after last follow-up
Stage Ⅰ-Ⅱ
Stage Ⅰ- Ⅲ
Stage Ⅲ
B
Fig S3: Chitinase activity was association with the number of liver metastases in CRC patients with synchronous metastasis.

## Slide 4
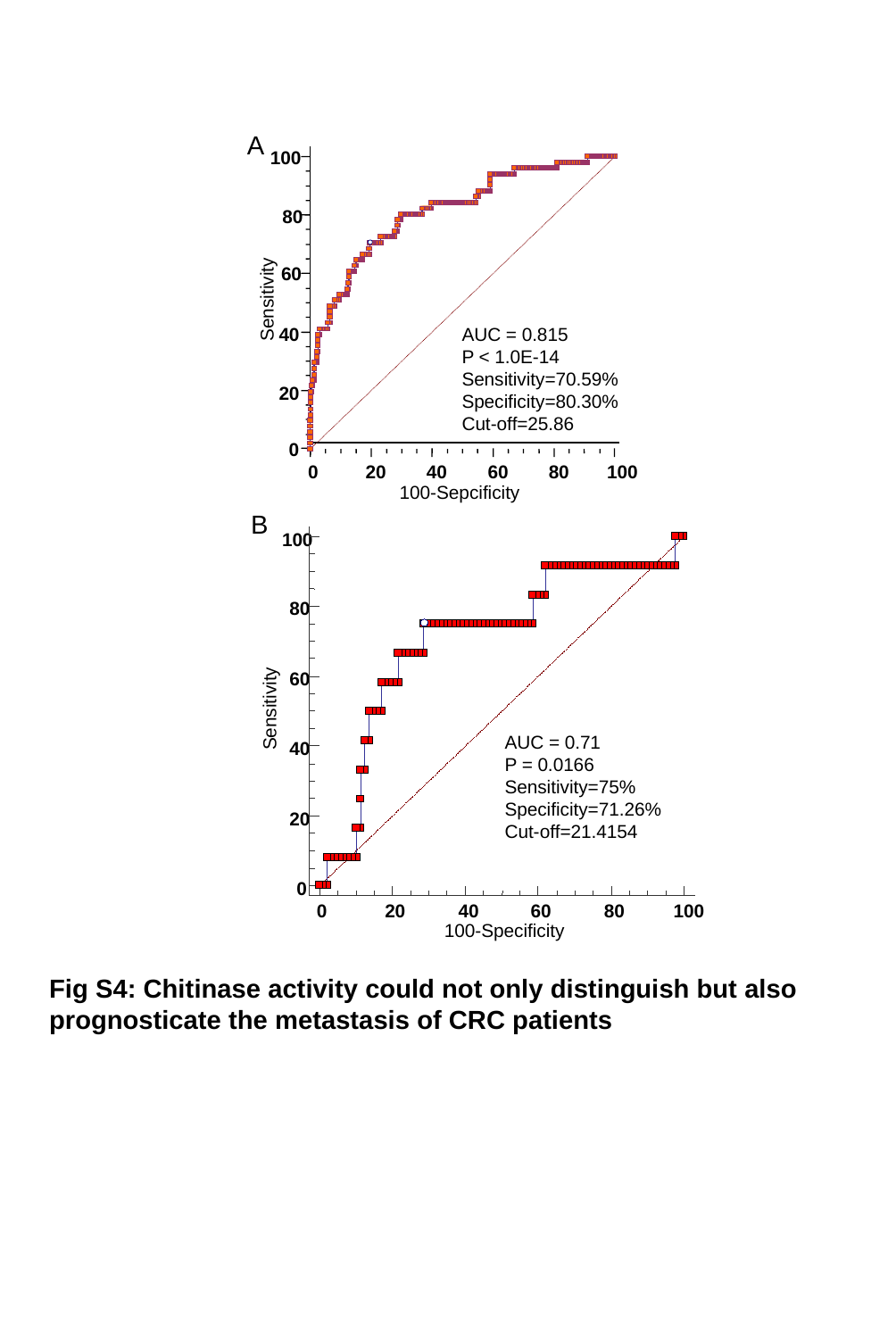

A
100
80
60
Sensitivity
40
20
0
0
20
40
60
80
100
100-Sepcificity
AUC = 0.815
P < 1.0E-14
Sensitivity=70.59%
Specificity=80.30%
Cut-off=25.86
B
100
80
60
Sensitivity
AUC = 0.71
P = 0.0166
Sensitivity=75%
Specificity=71.26%
Cut-off=21.4154
40
20
0
0
20
40
60
80
100
100-Specificity
Fig S4: Chitinase activity could not only distinguish but also prognosticate the metastasis of CRC patients

## Slide 5
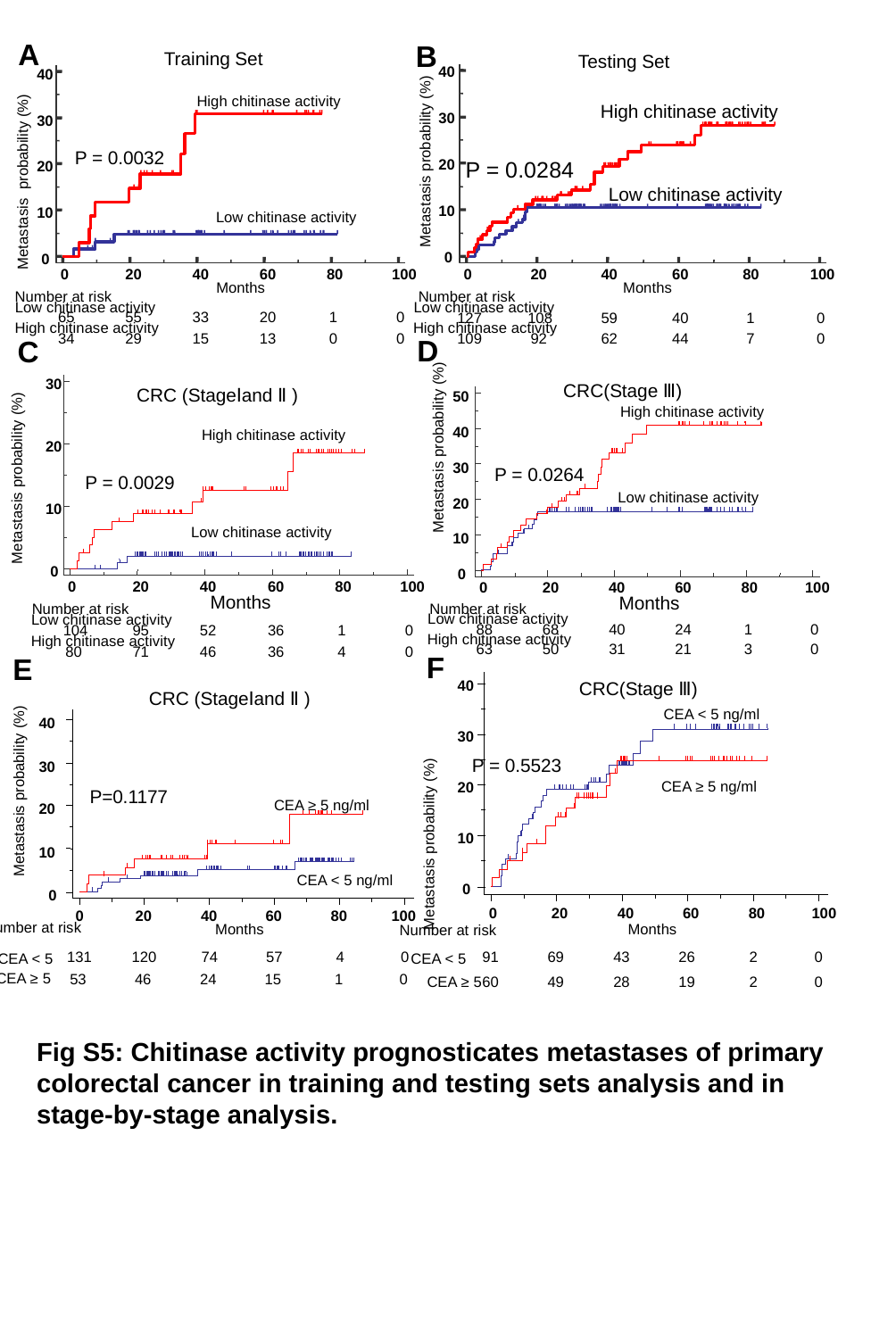

A
B
Training Set
Testing Set
40
30
Metastasis probability (%)
20
10
0
0
20
40
60
80
100
Months
Number at risk
Low chitinase activity
127
108
59
40
1
0
High chitinase activity
109
92
62
44
7
0
High chitinase activity
P = 0.0284
Low chitinase activity
40
30
20
10
0
0
20
40
60
80
100
Months
Number at risk
Low chitinase activity
65
55
33
20
1
0
High chitinase activity
34
29
15
13
0
0
High chitinase activity
P = 0.0032
Metastasis probability (%)
Low chitinase activity
C
30
20
Metastasis probability (%)
10
0
0
20
40
60
80
100
Months
Number at risk
Low chitinase activity
104
95
52
36
1
0
High chitinase activity
80
71
46
36
4
0
CRC (StageⅠand Ⅱ )
High chitinase activity
P = 0.0029
Low chitinase activity
D
CRC(Stage Ⅲ)
High chitinase activity
P = 0.0264
Low chitinase activity
50
40
30
 Metastasis probability (%)
20
10
0
0
20
40
60
80
100
Months
Number at risk
Low chitinase activity
88
68
40
24
1
0
High chitinase activity
63
50
31
21
3
0
F
40
CEA < 5 ng/ml
30
P = 0.5523
CEA ≥ 5 ng/ml
20
10
Metastasis probability (%)
0
0
20
40
60
80
100
Months
Number at risk
91
69
43
26
2
0
CEA < 5
60
49
28
19
2
0
CEA ≥ 5
CRC(Stage Ⅲ)
E
CRC (StageⅠand Ⅱ )
40
30
Metastasis probability (%)
P=0.1177
CEA ≥ 5 ng/ml
20
10
CEA < 5 ng/ml
0
0
20
40
60
80
100
Number at risk
Months
131
120
74
57
4
0
CEA < 5
CEA ≥ 5
53
46
24
15
1
0
Fig S5: Chitinase activity prognosticates metastases of primary colorectal cancer in training and testing sets analysis and in stage-by-stage analysis.
